# Building climate resilience, social sustainability and equity in global fisheries

**DOI:** 10.1038/s44183-023-00017-7

**Published:** 2023-08-07

**Authors:** Raul Prellezo, José María Da-Rocha, Maria L. D. Palomares, U. Rashid Sumaila, Sebastian Villasante

**Affiliations:** 1 AZTI, Marine Research, Basque Research and Technology Alliance (BRTA). Txatxarramendi Ugartea z/g, Sukarrieta - Bizkaia, Spain; 2 ITAM. Centro de Investigación Económica (CIE), Av. Camino Santa Teresa 930 C.P. 10700, CDMx, Mexico; Universidade de Vigo. Facultade de Ciencias Empresariais e Turismo, As Lagoas, Campus Universitario, 32004 Ourense, Spain; 3 ECOSOT, Department of Economic Theory, Universidade de Vigo, 36200 Vigo, Spain; 4 Sea Around Us Research Unit, Institute for the Oceans and Fisheries, University of British Columbia, Vancouver, BC Canada; 5 Fisheries Economics Research Unit, Institute for the Oceans and Fisheries and the School of Public Policy and Global Affairs, Vancouver, BC Canada V6T 1Z4 Canada; 6 EqualSea Lab-CRETUS, Department of Applied Economics, University of Santiago de Compostela, Santiago de Compostela, Spain

**Keywords:** Environmental sciences, Ocean sciences, Social sciences, Economics

## Abstract

Although the Paris Agreement establishes targets to limit global warming—including carbon market mechanisms—little research has been done on developing operational tools to achieve them. To cover this gap, we use CO_2_ permit markets towards a market-based solutions (MBS) scheme to implement blue carbon climate targets for global fisheries. The scheme creates a scarcity value for the right to not sequester blue carbon, generating an asset of carbon sequestration allowances based on historical landings, which are considered initial allowances. We use the scheme to identify fishing activities that could be reduced because they are biologically negative, economically inefficient, and socially unequitable. We compute the annual willingness to sequester carbon considering the CO_2_e trading price for 2022 and the social cost of carbon dioxide (SC-CO_2_), for years 2025, 2030 and 2050. The application of the MBS scheme will result in 0.122 Gt CO_2_e sequestered or US$66 billion of potential benefits per year when considering 2050 SC-CO_2_. The latter also implies that if CO_2_e trading prices reach the 2050 social cost of carbon, around 75% of the landings worldwide would be more valuable as carbon than as foodstuff in the market. Our findings provide the global economy and policymakers with an alternative for the fisheries sector, which grapples with the complexity to find alternatives to reallocate invested capital. They also provide a potential solution to make climate resilience, social sustainability and equity of global fisheries real, scientific and practical for a wide range of social-ecological and political contexts.

## Introduction

Climate change is negatively affecting marine biodiversity, and therefore, ecosystem services such as food provision is reduced^1,2^. The Paris Agreement adopted by the 2015 United Nations Climate Change Conference (PA) targets limiting global warming to 1.5–2 °C relative to the preindustrial level to avoid an irreversible loss in human wellbeing^3^. Article 6 of the PA establishes provisions for engaging in international cooperation through carbon market mechanisms, to support the achievement of nationally determined contributions. In global fisheries, the benefits of meeting the global warming targets are recognised, acknowledging that the increase in mean global temperature may lead to a potential decrease in fisheries catches^4^. Furthermore, 75% of maritime nations would benefit from these temperature targets, and 90% of the increase in catch potential, if climate targets are met, would occur within the territorial waters of developing countries^5^.

Carbon sequestration is defined as the near-permanent storage of carbon in a given area. Most studies focus on emissions management towards a reduction of fuel use by fishing fleets^6–8^. Worldwide fishing activity was estimated to produce in 2011 0.179 Gt of carbon dioxide equivalent (CO_2_e) or 2.2 CO_2_e per landed kg of fish^7^, values that have grown by 28% between 1990 and 2011^7^. Other studies consider the ocean carbon fluxes produced by the carcasses of large marine fishes^9^ or the comparison made between preindustrial and current fisheries^10^. These studies conclude that fishing activities have important effects on both the ocean’s health and carbon sequestration, and that it is also socially desirable to reduce them.

This paper focuses on the fish carbon mechanism: the uptake of atmospheric carbon into the ocean facilitated by marine vertebrates and the transport of carbon from the ocean surface to deep waters and sediments. Marine vertebrates store carbon in the ocean as biomass throughout their natural lifetimes, with larger individuals storing proportionally greater amounts over prolonged timescales^9^. The work presented here concentrates on the full biomass carbon storage of all marine fishes (i.e. blue carbon).

Little research has been done in developing universal operational tools to reach the benefits of achieving the PA climate targets. This paper addresses this issue, showing how the provisions of the PA can be stepped up by using a Market-Based Solution (MBS)^11,12^, that is, the opportunity cost of producing one ecosystem service (food provisioning) through its effect on carbon sequestration (climate regulation)^13^. The MBS scheme of this paper builds a supply curve which represents the willingness to reduce fishing, derived from the shadow prices created by entering fisheries into a carbon trade mechanism. It is based on an economic logic of inducing a scarcity value for the right to fish, creating an asset in the form of carbon allowances. This shadow price comes from imposing a price on the fishing fleet’s 'de facto' blue carbon initial allowances, calculated as the carbon stored in the fish landed over time. That is, an explicit opportunity cost of fishing from the climate targets perspective is created, although any other trade-off could be considered (e.g. nutritional properties of food or any other ecosystem service). This work builds on computing the fish withdrawal price at which fishing fleets have neutral decisions on whether to fish or trade their blue carbon allowances. For first-sale prices lower than the withdrawal price, fishing fleets will decide not to go fishing, but rather, trade their carbon allowances in the market. The reverse is true for first-sale prices higher than the withdrawal price.

To provide a reference of the scale at which the mechanism could support the PA targets, the European Union (EU) Emissions Trading System (ETS) is used. Set up in 2005, the system is a major EU policy to combat the impacts of climate change, and the world’s first major carbon market with around three-quarters of global carbon trade^14^. This system works by setting up a 'cap and trade' mechanism, where a total amount of certain greenhouse gases is allowed to be emitted each year, while companies receive, buy, and sell emission allowances. However, carbon markets seldom reflect the full social cost of production and therefore, the Social Cost of Carbon dioxide (SC-CO_2_) is also used as an additional reference. SC-CO_2_ is defined as the monetised value of the damages to society caused by an incremental tonne of CO_2_^15^. Calculations are based on CO_2_ prices that reflect the SC-CO_2_ of US$543tCO_2_e^−1^ for 2050, US$203 tCO_2_e^−1^ for 2030 and US$165 tCO_2_e^−1^ for 2025^16^. These prices reflect the social cost of limiting global warming to 2.5 °C relative to the preindustrial level considering a cap for an average of 100 years.

Beyond efficiency, economic inequalities are among the most pressing challenges of our times^17^. Furthermore, disagreement over the equity principles persists^18^. Therefore, in this paper, we test whether the MBS proposed suggests distributional effects of the ocean benefits, by describing the countries favoured or not, and computing the overall equity change of these benefits before and after implementing the MBS. The paper shows how it is possible to reallocate fishing activities with a social and/or market(s) negative balance. Therefore, the study concludes that the inclusion of the fishing industry in a carbon trading scheme, considering in a non-exclusive way the blue carbon concept, induces a more climate-resilient, socially efficient and equitably balanced fishing activity.

## Results

### Global results of the application of the MBS

The mean reported landings for the industrial and artisanal fisheries in Exclusive Economic Zones (EEZ) of maritime countries and in the high seas for the mean of the period 2011–2018 are estimated at 0.106 Gt (US$222 billion in value) per year. Anchoveta (*Engraulis ringens*), marine fishes not identified and Alaska-pollack (*Theragra chalcogramma*) are the most landed species in this period, representing 11, 10 and 4% of the total landings, respectively.

We estimate that total landings represent a blue carbon budget of 0.161Gt CO_2_e yr^−1^ (the 'cap' or initial allowances), or between US$11 billion yr^−1^ (at 2022 ETS prices) to US$88 billion yr^−1^ (at 2050 SC-CO_2_ prices) in potential benefits. Artisanal fisheries represent one-fourth of the global landings and value, while 3% of global landings were made on the high seas.

**Fig. 1:**
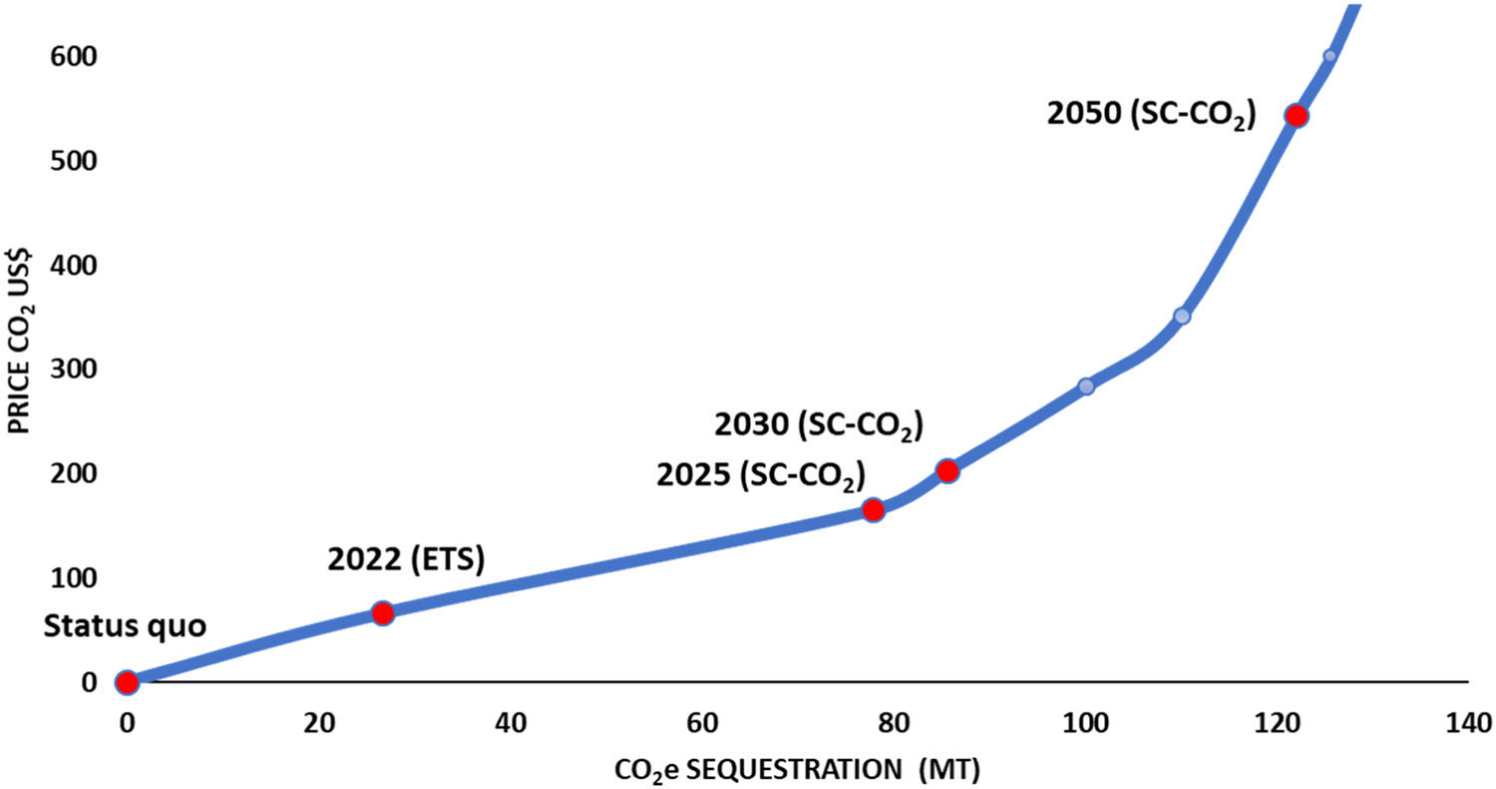
Global supply curve of blue carbon sequestration. Based on the CO_2_e trading price for 2022 (ETS) and the Social Cost of Carbon (SC-CO_2_), for the years 2025, 2030 and 2050.

When the trading scheme is applied, Fig. 1 (blue line) shows the CO_2_e supply curve for global fisheries if the scheme would be applied. The CO_2_e sequestered increase, moving from zero if the CO_2_e trading price is zero (there is no opportunity cost for fishers) to 0.027 Gt (17% of the total CO_2_e 'cap') per year if 2022 ETS prices (US$66 tCO_2_e^−1^) are used. The application of this proposed scheme will result in 0.122 Gt CO_2_e yr^−1^ sequestered (76% of the CO_2_e 'cap') or US$66 billion yr^−1^ of potential benefits when considering 2050 SC-CO_2_. The latter also implies that if CO_2_ trading prices reach the 2050 SC-CO_2_, around 75% of the landings worldwide would be more valuable as carbon than as foodstuff in the market.

### Results by EEZ, and major fishing areas of the application of the MBS

**Fig. 2:**
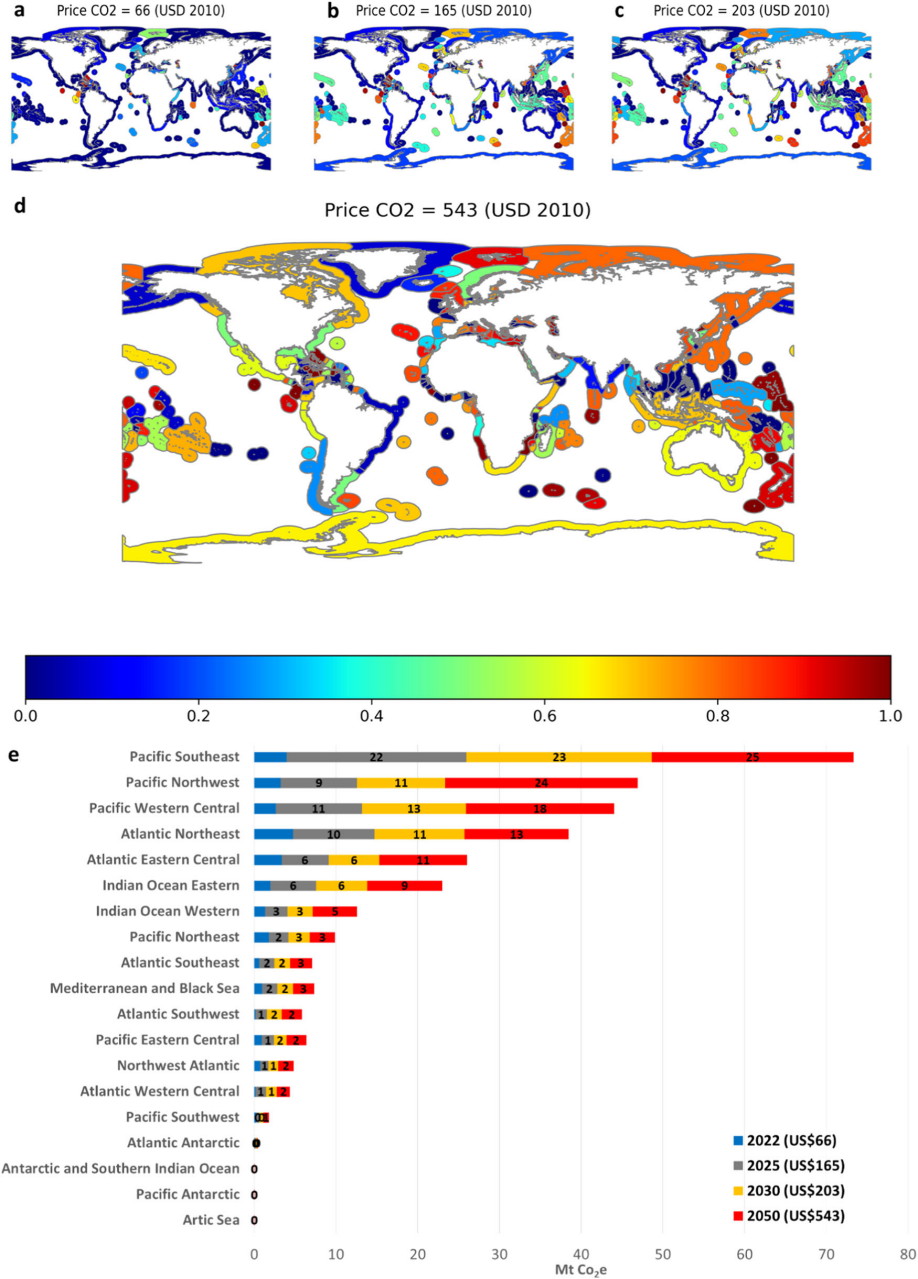
Global CO_2_e removals under different scenarios for the EEZs, high seas, and FAO major fishing areas. CO_2_e removals in percentage from the status quo situation (mean 2011–2018) under different prices for CO_2_ by EEZ (0 -dark blue- implies 0% of CO_2_e removals relative to the status quo, and 1 -dark red- implies 100% of CO_2_e removals compared to the status quo). ETS 2022 exchange prices (**a**); SC-CO_2_ to meet the PA in 2025 (**b**); SC-CO_2_ to meet the PA in 2030 (**c**); SC-CO_2_ to meet the PA in 2050 (**d**); CO_2_-eq removals in million tonnes by FAO major fishing areas of a total of 0.027 Gt yr^−1^ in 2022, 0.078 Gt yr^−1^ in 2025, 0.87 Gt yr^−1^ in 2030 and 0.122 Gt yr^−1^ in 2050, respectively (**e**).

Figure 2 shows the global CO_2_e removals considering 2022 ETS (US$66 tCO_2_e^−1^) (Fig. 2a) 2030 ETS (US$203 tCO_2_e^−1^) (Fig. 2b) 2050 ETS (US$165 tCO_2_e^−1^) (Fig. 2c) for the EEZs, high seas, and Food and Agriculture Organisation (FAO) major fishing areas (Fig. 2e). This last is an ad hoc division of sea boundaries defined by FAO determined on various considerations with consulting international fishery agencies. EEZs prescribed by the 1982 United Nations Convention on the Law of the Sea, is an area of the sea in which a sovereign state has exclusive rights regarding the exploration and use of marine resources and stretches from the outer limit of the territorial sea out to 200 nautical miles from the coast of each state. Finally, high seas are defined as all parts of the mass of saltwater surrounding the globe that are not part of the territorial sea or internal waters of a state.

Results suggest that the main contributors in landings and CO_2_e removals are Peru (12 and 13% of total landings and CO_2_e removals, respectively) and China (9% each). Peru’s results are driven by their volume of anchoveta landings (91% of the landings of this species are made in Peru’s EEZ), while China’s results are driven by their total landings share worldwide (12% of the world’s landings in volume) with a more diverse landings portfolio than Peru.

### Distributional effects of the application of the MBS

Our results reflect the fact that the spatial distribution of the global volume of landings, landed value and possibilities of CO_2_ sequestration are not fully correlated. Therefore, the total landings would not be equally shared among different EEZs. For example, considering carbon exchange prices in 2022, no landings would be removed from the Arctic Sea, the Antarctic, and the Southern Indian Ocean, while 52% of the Pacific Northeast landings would be more valuable as carbon than as a foodstuff. If the SC-CO_2_ to meet the 2050 climate target is considered, the Arctic Sea would present the lowest value in terms of landings removal (2%), while in the Atlantic Northeast, 92% of landings would be more valuable as carbon than as foodstuff (Fig. 2e).

At 2022 carbon exchange prices, the opportunity cost of not using this scheme is estimated at US$0.8 billion yr^−1^ (0.3% of total landings value). However, when different SC-CO_2_ are considered, the opportunity cost would increase to US$6.7 billion yr^−1^, US$10 billion yr^−1^ and US$49 billion yr^−1^ to achieve the targets of the PA in 2025, 2030 and 2050, respectively (representing 3, 4.5 and 22% of the total landings value). The economic efficiency of the scheme proposed here would also be higher compared to the current status quo of the management of global fisheries. The average price with the scheme in place should be, in 2022, 0.3% higher (US$2109 t^−1^) than in the status quo (US$2102 t^−1^), while when the 2050 climate target is considered, the price would be 22% higher than in the status quo in real terms (US$2567 t^−1^) and considering the status quo landed quantity.

At 2022 exchange carbon prices (US$66 tCO_2_e^−1^), the global application of the scheme suggests that of the total landing removals from the oceans, 1.7% would come from landings of artisanal fishing fleets and the rest from industrial fleets. At this carbon exchange price, 1.1% of the initial carbon allowances for artisanal fleets would be additionally sequestered, while 21% would be sequestered from industrial fishing fleets. Considering the 2050 climate target, the carbon allowances additionally sequestered would rise to 26 and 92% for artisanal and industrial fleets, respectively. In the high seas, landings removals at 2022 exchange prices of CO_2_ permits would be 5% (≈ in CO_2_e). In addition, considering climate targets for 2025, 2030 and 2050, landing removals would be 20% (18% in CO_2_e), 21% (20% CO_2_e) and 60% (59% CO_2_e), respectively.

Our results show that at 2022 exchange carbon prices pelagic trawlers would be the most affected fishing gear, with a reduction of 50% of their status quo (mean 2011–2018) landings; followed by hand lines (41% reduction), encircling nets (24%), purse seiners (21%) and harpoons (19%). If the carbon trading prices reach the 2050 SC-CO_2_, the landing removals of these fishing gears will be, overall, around 90% of their status quo landings.

**Fig. 3:**
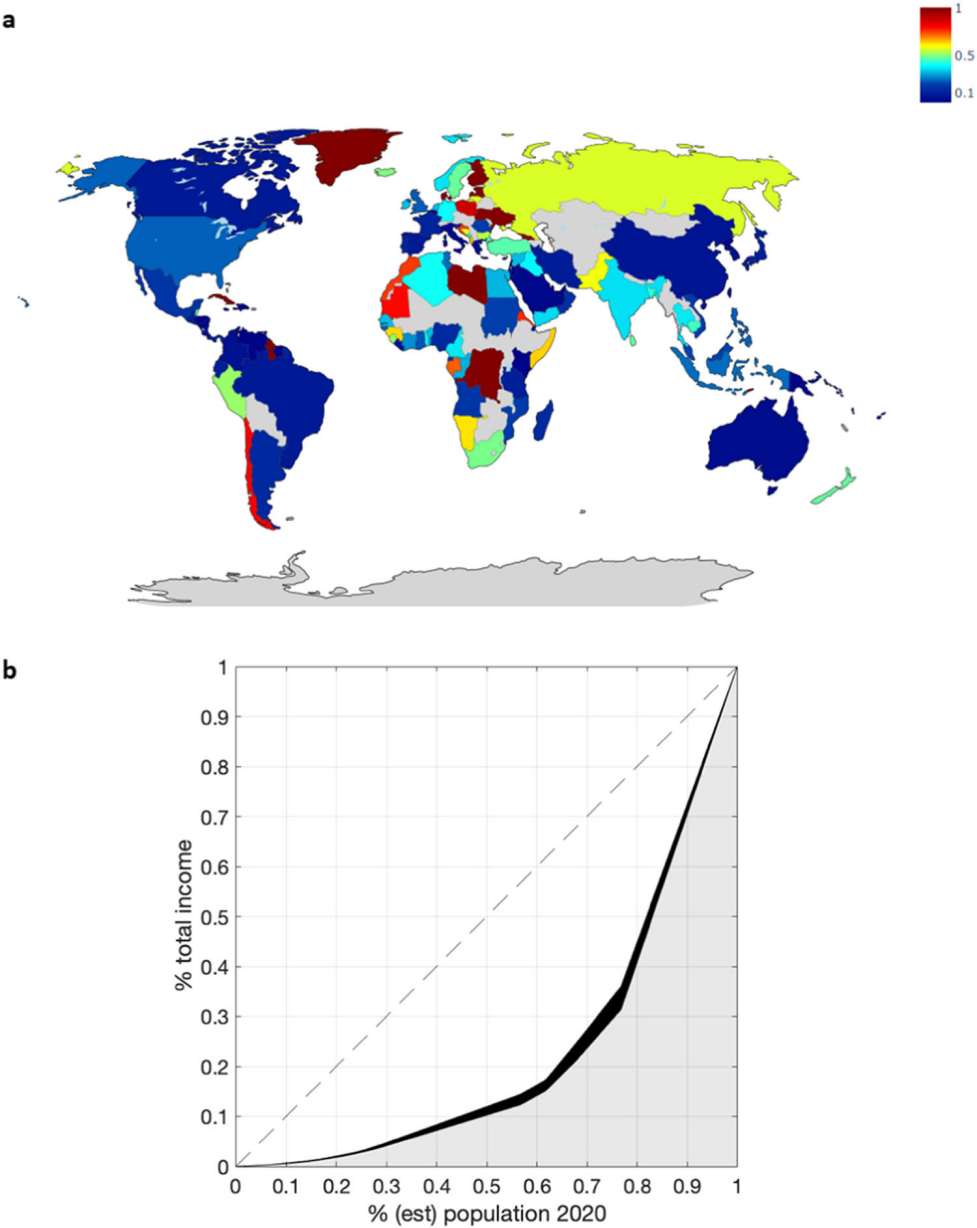
Distributional benefits of the MBS for global fisheries. **a** Changes in the distribution of the global fisheries income by countries in 2020 compared to the status quo situation if the mechanism would be applied considering the SC-CO_2_ in 2050 (0 -dark blue- implies 0% of increase in income per capita relative to the status quo, and 1 -dark red- implies 100% of increase in income per capita relative compared to the status quo), **b** the Lorenz curve coefficients in the status quo situation (Gini = 0.56024), and the area gained (black-shadowed) with the mechanism in place (SC-CO_2_ = US$543 tCO_2_e^−1^, Gini = 0.52793) considering the estimated 2020 population.

This MBS also suggests positive distributional effects of the ocean benefits. Our results show that, at the EEZ level, Cape Verde, Guadeloupe (France), Faroe Islands and Greenland (Denmark), and Madeira Islands (Portugal) would be the main beneficiaries from the system (for all carbon prices above the 2022 ETS price), while Turks and Caicos Islands (UK), Bahamas, Antigua and Barbuda and North Cyprus would not experience changes after the implementation of the MBS. It is also remarkable that in the case of Finland, the CO_2_e sequestered would be 80, 95, 99 and 99% of its initial allowance for 2022 (ETS), 2025, 2030 and 2050 SC-CO_2_, respectively. Greenland (Denmark), Russian Federation (Baltic) and Sweden (Baltic) also present similar results as Finland (Fig. 3a) (the Supplementary Material includes a list of CO_2_e additionally sequestered and landings removals, by EEZ and FAO major fishing area for all countries in the world).

We also constructed Lorenz concentration curves and computed Gini coefficients to illustrate how the MBS generates changes in the distribution of landings value and the income inequalities^19^. The inclusion of the fisheries sector in this scheme would reduce the Gini coefficient from 0.560 (status quo) to 0.559 in 2022, or even more to 0.527 in 2050, which would imply a higher equality in the income distribution of ocean benefits (Fig. 3a). Currently, 20% of the world’s population accounts for 60% of the fishing landing’s income, while with the scheme in place and considering the 2050 climate targets, this 20% would account for 48% of the landing’s income (Fig. 3b). The main regions benefiting in 2050 would be Northern Africa and Western Asia (44% increase in total income compared to the status quo), Central and Southern Asia (36%), and Sub-Saharan Africa (33%).

## Discussion

The results presented above reveal that the average carbon content is set at 1.51 kg CO_2_e per landed kg, considering the mean of the period 2011–2018. However, this must be considered a lower bound, given that the research has been limited only to the blue carbon content of fish. It excludes other active biological mechanisms such as the biological pump^20^, or the effect of several fishing gears on the disturbance of seabed carbon stores that can re-mineralise sedimentary carbon to CO_2_^21,22^.

The paper proposes the internalisation of shadow prices for harvested fishes calculated through their blue carbon content, while economic efficiency is obtained by allowing the trade of CO_2_e allowances. This scheme provides the global economy with an alternative for the fisheries sector, which grapples with the complexity to find alternatives to reallocate invested capital. It also induces reducing (over)fishing and contributes to build climate resilience and a more equitable distribution of income from the oceans. The internalisation of the climate effect of fisheries (considering only the blue carbon) would imply a 22%-increase in the average ex-vessel prices worldwide if 2050 climate targets are considered.

The developed scheme is in accordance with the need to integrate other alternative economic paradigms, such as degrowth economics already proposed for land-based food systems^23^. Moreover, it does not compete with other fisheries management systems currently in place (e.g. Marine Protected Areas^24^), nor with other nature-based solutions. Although not tested here, higher future biomasses from current lower landings could also be relevant. Larger fish stocks usually increase economic profits^25^. However, the MBS scheme proposed here is independent of the amount of fishes in the oceans and does not imply that this dynamic effect should be converted, *per se*, into higher landings. On the contrary, the induced landing reduction would also support an increase in the resilience in the oceans^26^ and society would obtain another value from ecosystem services beyond protein provision and climate regulation.

In this paper, estimates using SC-CO_2_ are based on a temperature increase limit of 2.5 °C compared to the preindustrial level and considering a cap for an average of 100 years^15,16^. Therefore, the effect of the MBS scheme would be even higher (in carbon sequestration and landings removals) if the temperature constraint would be set to 1.5–2 °C (PA), or if the SC-CO_2_ would be calculated using a hard cap for a single period. This implies that results should be interpreted more as a reference to the possibilities that this scheme offers of internalising the trade-offs rather than considering the absolute values we have obtained. Furthermore, the values for SC-CO_2_ are subject to revisions as new improved probabilistic socioeconomic projections, climate models, damage functions, and discounting methods that collectively reflect theoretically consistent valuation of risk, come in refs. ^27,28^.

Although the scheme developed here would help in mitigating the climate change-driven global fisheries revenues losses by 2050^29^, market-based mechanisms’ success is usually related to how economic efficiency gains are shared^18–20^ and how fishers are adequately compensated for the transition costs^29^. While the latter is guaranteed by the option that fishers have of exerting the right to fish or trading the carbon allowances, we show how equity will be improved at spatial and temporal scales favouring those fishing areas of the world generating lower incomes relative to those with higher ones.

We recognise the complexity of the operationalization of a global CO_2_ trading system, and there are, of course, challenges to be addressed. First, the fishing sector is not currently under any carbon trading system. It is complex to reach a global compulsory scheme with all the parties involved, because there is always the threat of the free-riding problem^29^. Second, even if each tonne of carbon sequestered should have the same value on the global level, a national manager could adopt a different value for each tonne of carbon sequestered^30^. Third, it is still unclear if the carbon trading prices will reach the social cost of it.

However, the potential gains of an MBS scheme -as the one proposed here- are not only reflected in terms of the economic efficiency of the fishing activities. We have shown that it also generates a more equitable distribution of the income obtained from marine resources. Furthermore, this gain is gradual, and at 2022 ETS prices, the effect is higher and more intense in the redistribution of income than at the efficiency level. In summary, it produces a socialisation of the climate costs of fishing and benefits the overall fisheries challenge, which is to keep global ocean biomass high enough to keep a profitable fisheries sector, while at the same time increasing resilience which supports other values that we obtain from the seas.

## Methods

### Global fisheries data

To illustrate the potential global benefits of the scheme, the *Sea Around Us* (SAU^31^) dataset of reconstructed catches by artisanal and industrial fishing sectors and prices (in 2010 US$) of this catch for the period 2011–2018 was used. Catches were summarised by FAO major fishing areas, EEZs, high seas and fishing countries, by fleet and gear used, at the species, genus and family level. Catch discarded at sea (carbon is not extracted but returned to the sea), recreational and subsistence landings were excluded from the analysis.

### Blue carbon estimates by species

The carbon content by species was obtained from ref. ^32^. Species without carbon content information were assigned the mean carbon content of species in the lowest taxonomic level (family or genus).

### Computing the market-based solution scheme

The withdrawal price was calculated as the price where society is indifferent in the valuation of the fish as foodstuff or as carbon as follows:

1 $$\begin{array}{l}Withdrawal\,pric{e}_{S,A,EEZ,Y,FS,FG}=\left(\right.\left(\right.Market\,Pric{e}_{S,A,EEZ,Y}\\\ast Landing{s}_{S,A,EEZ,Y,FT}\left.\right)/Carbo{n}_{S}\left.\right)\left.\right)\ast Facto{r}_{FS}\end{array}$$

Where *S* stands for the species; *A*, denotes the major FAO fishing area; *EEZ* indicates the economic exclusive zone; *Y* denotes the year; *FS* denotes the fishing sector (artisanal or industrial); and *FG* shows the fishing gear. *Factor* in Eq. 1 means the value of 1 for artisanal fleets, which considers that value added equals the landing value. For computing purposes only, “High Seas” are treated as another EEZ.

For the industrial fleets, only the proportion of the profits which are considered normal (sufficient revenues to cover its total costs and remain competitive in an industry), and not extra normal (where the profits exceed these levels) were considered. To calculate this proportion, we took the ratio of the normal profits (subtracting from the market value of the landings, the crew payments and the rental price of capital) to the landings. For reference, we used the net profit/value of landings for the EU fishing fleet, one of the most important fishing fleets worldwide^33^. The value was 0.14, and is the one used in the main text results. The rationale behind utilising a factor of 1 for artisanal fishing and 0.14 for industrial fishing is based on solely compensating the remuneration obtained by the owner of capital (profits). The concept of profits (and rent) primarily applies to the industrial sector, while the relationship between capital and labour compensation is blurred in the case of artisanal fishing. Empirical research has shown that the development of artisanal fisheries is not associated with return on capital investment^34^. Nevertheless, a sensitive analysis to different values and options to this factor is provided in the section below.

A comparison algorithm was created in R^35^, producing positive landing removals when the withdrawal price was lower than the price of each CO_2_e price, for each entry of the dataset defined by the species, year, FAO area, EEZ, year, fishing sector and fishing gear.

### Computing the inequality index

Inequality has been analysed by computing the Lorenz curve and Gini index^36^, of the income from landings (status quo) or landings plus income from trading the CO_2_e. (when the MBS mechanism is in place) for each countries’ population based on the data provided by the UN^37^).

To calculate the change in the average price worldwide required to internalise the climate effect of fisheries (considering only the blue carbon), the value of landings of the period 2011–2018 was compared to the total value of landings plus the mechanism under different carbon prices. Both calculations were then divided by the average landings of the period 2011–2018 to obtain the mean price without considering the mechanism and the average price considering it.

### Sensitivity analysis

A sensitive analysis of the initial allowances, in the form considered here (average landings of the period 2011–2018) was also performed. Regarding the initial cap, it provides a standard error of the mean of 0.106 ± 0.0083 Gt yr^−1^ (US$222 ± 0.0143 billion yr^−1^ in value). In addition, the carbon removals considering the standard error would be 0.027 ± 0.0007 Gt yr^−1^ if 2022 ETS prices (US$66 tCO_2_e^−1^) were used, and 0.122 ± 0.0023 Gt yr^−1^ in the case of 2050 SC-CO_2_ (US$543 tCO_2_e^−1^) (for more details, see Table 1 in the Supplementary Material).

For reference, we used the net profit/value (0.14) of landings for the EU fishing fleet. Nevertheless, as many other countries do not provide economic data, this assumption cannot be generalised worldwide and therefore, we also provide results for an industrial factor of 0.33 (based on the general economy capital share^38^) and of 1 (same as for the artisanal fishing fleets).

The main result is that the higher the factor for the industrial fisheries, the lower the landings removals (and therefore, the lower the carbon sequestered) will be. In addition, the distribution of these removals are more affected by the lower carbon price considered. Furthermore, for the 2050 carbon price, the removals’ distribution tends to converge (for more details, see Supplementary Table 1).

We also computed the mechanism considering that artisanal fisheries are treated equally as industrial ones (factor 0.14 for both). In this case total CO_2_e additionally sequestered removals will increase from 0.027 Gt yr^−1^ to 0.034 Gt yr^−1^ (+29%) if 2022 ETS prices (US$66 tCO_2_e^−1^) were considered and from 0.122 Gt yr^−1^ to 0.122 (+0.147%) Gt yr^−1^ if 2050 SC-CO_2_ (US$543 tCO_2_e^−1^) were considered. In this case, all the additional CO_2_e sequestration will come as a result of artisanal fisheries landings’ reduction (for more details, see Supplementary Table 1).

### Supplementary Information

Table 1_Supplementary Material

## Data Availability

The datasets generated during the current study are available in the repository BCR available at https://github.com/rprellezo22/BCR. Original data can be accessed via this link https://www.seaaroundus.org/data/#/eez sourced from the Sea Around Us dataset.

## Code availability

The underlying code for this study is available in the repository BCR and can be accessed via this link https://github.com/rprellezo22/BCR.
